# Submicroscopic subtelomeric aberrations in Chinese patients with unexplained developmental delay/mental retardation

**DOI:** 10.1186/1471-2350-11-72

**Published:** 2010-05-11

**Authors:** Ye Wu, Taoyun Ji, Jingmin Wang, Jing Xiao, Huifang Wang, Jie Li, Zhijie Gao, Yanling Yang, Bin Cai, Liwen Wang, Zhongshu Zhou, Lili Tian, Xiaozhu Wang, Nan Zhong, Jiong Qin, Xiru Wu, Yuwu Jiang

**Affiliations:** 1 Department of Pediatrics, Peking University First Hospital, Beijing, China; 2 Department of Neurology, Beijing Children's Hospital, Capital Medical University, Beijing, China; 3 Shanxi Medical University, Taiyuan, China; 4 Department of Neurology, Capital Institute of pediatrics Affiliated Children's Hospital, Beijing, China; 5 CapitalBio Corporation-National Engineering Research Center for Beijing Biochip Technology, Beijing, China; 6 Department of Pediatrics, China-Japan Friendship Hospital, Beijing, China; 7 Department of Pediatrics, Xuanwu Hospital, Capital Medical University, Beijing, China; 8 Peking University Center of Medical Genetics, Beijing, China

## Abstract

**Background:**

Subtelomeric imbalance is widely accepted as related to developmental delay/mental retardation (DD/MR). Fine mapping of aberrations in gene-enriched subtelomeric regions provides essential clues for localizing critical regions, and provides a strategy for identifying new candidate genes. To date, no large-scale study has been conducted on subtelomeric aberrations in DD/MR patients in mainland China.

**Methods:**

This study included 451 Chinese children with moderate to severe clinically unexplained DD/MR. The subtelomere-MLPA (multiplex ligation dependent probe amplification) and Affymetrix human SNP array 6.0 were used to determine the subtelomeric copy number variations. The exact size and the breakpoint of each identified aberration were well defined.

**Results:**

The submicroscopic subtelomeric aberrations were identified in 23 patients, with a detection rate of 5.1%. 16 patients had simple deletions, 2 had simple duplications and 5 with both deletions and duplications. The deletions involved 14 different subtelomeric regions (1p, 2p, 4p, 6p, 7p, 7q, 8p, 9p, 10p, 11q, 14q, 15q, 16p and 22q), and duplications involved 7 subtelomeric regions (3q, 4p, 6q, 7p, 8p, 12p and 22q). Of all the subtelomeric aberrations found in Chinese subjects, the most common was 4p16.3 deletion. The sizes of the deletions varied from 0.6 Mb to 12 Mb, with 5-143 genes inside. Duplicated regions were 0.26 Mb to 11 Mb, with 6-202 genes inside. In this study, four deleted subtelomeric regions and one duplicated region were smaller than any other previously reported, specifically the deletions in 11q25, 8p23.3, 7q36.3, 14q32.33, and the duplication in 22q13. Candidate genes inside each region were proposed.

**Conclusions:**

Submicroscopic subtelomeric aberrations were detected in 5.1% of Chinese children with clinically unexplained DD/MR. Four deleted subtelomeric regions and one duplicated region found in this study were smaller than any previously reported, which will be helpful for further defining the candidate dosage sensitive gene associated with DD/MR.

## Background

Developmental delay/mental retardation (DD/MR) occurs in 1%-3% of the general population [1,2]. MR is defined as a significant impairment of both cognitive (IQ < 70) and social adaptive functions, with onset before 18 years of age. MR can not be diagnosed until the child is older than 5 years, when the intelligence measurements are reliable. For children younger than 5 years, the term "DD" is usually used. The etiological diagnosis is challenging, because diverse genetic and environmental factors may contribute to its pathogenesis [3,4]. It is estimated that 25%-50% of moderate to profound DD/MR is resulted from genetic etiology [5]. G-banding karyotyping is a routine clinical test for DD/MR patients, and the reported frequency with which it detects microscopic chromosomal aberrations varies between 9%-36% [6]. However, microscopic techniques cannot detect interstitial or terminal subtelomeric microdeletions and microduplications [4]. Progress in molecular cytogenetic techniques, such as fluorescent in situ hybridization (FISH), multiplex ligation dependent probe amplification (MLPA) and array comparative genomic hybridization (aCGH), has resulted in detection of submicroscopic subtelomeric rearrangements in approximately 5% (0-23%) of DD/MR patients [7-14].

Subtelomeric regions are usually enriched for genes, and are more susceptible to aberrant rearrangements than other chromosomal regions [15,16]. Subtelomeric imbalance is widely accepted as leading to DD/MR or multiple congenital anomalies (MCA), although the exact cause-and-effect relationship has not been well defined [17-24]. The clinical consequences are probably determined by the location and kind of the rearrangement, such as deletions or duplications, as well as the size of the aberrations, including the numbers and function of the genes involved [20]. These aberrant regions are likely to contain undiscovered candidate genes associated with DD/MR. Fine mapping of aberrant subtelomeric regions to determine the critical regions and genes has become a new strategy for identifying novel candidate genes for DD/MR [25].

In 2007, 11,820,000 people in mainland China had an intellectual disability, of whom 954,000 were younger than 6 years of age. To date, no large-scale study has been conducted on subtelomeric aberrations in Chinese DD/MR patients. Here we report the investigation of 451 Chinese children with clinically unexplained DD/MR, using subtelomere-MLPA and Affymetrix human SNP array 6.0. Subtelomeric aberrations were identified, their exact sizes were defined, and possible candidate genes are proposed.

## Methods

### Patients

Patients with unexplained DD/MR were defined as those without etiological diagnosis after thorough clinical evaluations, and were included based on the following criteria: 1) moderate to severe DD/MR (IQ < 55, assessed with Gesell Developmental Schedules or Wechsler intelligence scale for children); 2) definite exclusion of perinatal brain injury; 3) no history of toxication, hypoxia, central nervous system infection and cranial trauma; 4) normal routine karyotyping; 5) no evidence of recognizable inherited metabolic disorder or specific neurodegenerative disorders by brain imaging and blood/urinary metabolic screening; 6) negative for mutations in the *FMR1 *gene for male patients; 7) negative for typical clinical features of Rett syndrome for female patients.

All 451 subjects were Chinese children from the Department of Pediatrics in Peking University First Hospital and Department of Neurology in Beijing Children's Hospital, recruited from 2006-2008, and informed consent was obtained. Genomic DNA was extracted from peripheral blood for each index patient and his or her parents. The research was approved by Medical Ethics Committee of Peking University First Hospital.

### MLPA for screening of subtelomeric rearrangements

A specifically designed set of probes for testing subtelomeric imbalances in the SALSA P070 and P036B human telomere test kits (MRC-Holland, Amsterdam, Netherlands; http://www.mrc-holland.com) was used. For each patient with consistent positive results from both kits, their parents' samples were tested.

The MLPA mix contained probes for all subtelomeric regions except the short arms of the acrocentric chromosomes (13p, 14p, 15p, 21p and 22p), for which, probe recognition sequences were on the q arm, in one of the genes just proximal to the telomeric repeats. Sequences detected by two probes mixes were different from each other. MLPA analysis was performed following the manufacturer's instruction. Amplification products were identified and quantified by capillary electrophoresis on an ABI 3100 genetic analyzer. The fluorescent signal strength of the PCR products was determined using Genemarker1.5 software. For each patient, the normalized peak pattern of each subtelomeric region was divided by the average peak pattern of all samples (n > 10) in the same experiment. The resulting values were approximately 1.0 for wild type peaks, <0.75 for deletions, and >1.3 for duplications.

### Affymetrix human SNP array 6.0 assay

To confirm and accurately define the exact size of each subtelomeric aberration region found by MLPA, Affymetrix genome-wide human SNP array 6.0 was used. Each array has 1,800,000 genetic markers, including more than 906,600 single nucleotide polymorphisms (SNPs) and more than 946,000 probes for the detecting copy number variations (CNVs). The high density of the probes across the genome and combination of a SNP array with aCGH probes in a single chip enabled the accurate definition of the size of each aberrant region.

Digestion, ligation, PCR, labeling, hybridization and scanning were performed following standard protocols. Partek software (version 6.3) was used for the analysis of CNVs. Duplicated or deleted regions were determined by Hidden Markov Model (HMM) calculation. Involvement of at least three contiguous probe sets was required, and when a copy number calculated by HMM as <1.5 was considered a deletion, while >2.5 was considered a duplication.

## Results

Screening of subtelomeric rearrangements with MLPA revealed an abnormality in 34 of 451 patients. Deletion in a single region was found in 22 patients (15pter del in 5; 4pter del in 4; 13pter del in 3; 11qter del in 2; 9pter del in 2; and deletion in 1pter, 7qter, 8pter, 15qter, 16pter and 22qter in 1 patient each). Deletions in two different subtelomeric regions were identified in 2 patients (13pter del + 22qter del, 14qter del +17qter del). Duplication in single subtelomeric region was found in 3 patients (3qter dup in 2; 22qter dup in 1). 7 patients were found to have both a deletion and duplication (8pter del + 7pter dup in 2; 2pter del + 4pter dup; 10pter del + 6qter dup; 9pter del + 19pter dup; 7pter del + 12pter dup; and 6pter del + 8pter dup). Subsequent MLPA assay for parents showed that all rearrangements were absent in the parents.

To further confirm and accurately define the exact size of each subtelomeric aberration detected by MLPA, subsequent assay with Affymetrix genome-wide human SNP array 6.0 was performed on 24 patients. Because the MLPA probe recognition sequences for 13p and 15p are actually on the q arm, and do not represent the subtelomeric regions, 8/34 subjects with 15pter del or 13pter del were not studied further. Array analysis was also not performed for an additional 2 of the 34, specifically with 8pter del + 7pter dup, and 9pter del + 19pter dup, due to insufficiency of DNA samples.

Subtelomeric copy number aberrations were confirmed in 23 of 24 patients, with a final detection rate of 5.1% (23/451), of which 16 (69.6%) were found to have simple deletions, 2 (8.7%) had a simple duplication and 5 (21.7%) had complex rearrangements. The deletions involved 14 different subtelomeric regions (1p, 2p, 4p, 6p, 7p, 7q, 8p, 9p, 10p, 11q, 14q, 15q, 16p and 22q), and duplications involved 7 subtelomeric regions (3q, 4p, 6q, 7p, 8p, 12p and 22q). In 4p, 7p, 8p and 22q, both subtelomeric deletions and duplications were detected. The size of the deletions varied from 0.6 Mb to 12 Mb, with 5-143 genes inside. Duplicated regions were 0.26 Mb to 11 Mb, with 6-202 genes inside.

Clinical features of patients and the results from MLPA and Array are summarized in Table 1 and Table 2.

**Table 1:** The clinical characteristics of DD/MR patients with or without subtelomeric aberrations

Clinical Characteristics	Patient with subtelomeric imbalance confirmed by MLPA and Array (23 patients) N (%)	Patients without sub-telomeric aberrations (428 patients) N (%)
**Gender, M/F**	10/13	300/128

**Age**	1.9 y (6 m-7 y 10 m)	4.2 y (4 m-29 y)

**Seizure**	3 (13.0)	117 (27.3)

**Autistic features**	0 (0.0)	0 (0.0)

**Family history of DD/MR**	3 (13.0)	64 (15.0)

**Maternal history of miscarriages**	3 (13.0)	9 (2.1)

**Microcephaly**	11 (47.8)	104 (24.3)

**Macrocephaly**	0 (0.0)	17 (4.0)

**Low birth weight**	7 (30.4)	32 (7.5)

**Growth delay**	5 (21.7)	104 (24.3)

**Minor facial and non-facial dysmorphism**	18(78.3)	217 (50.7)

**Other congenital abnormalities**	9 (39.1)	63 (14.7)

**Table 2:** Clinical findings, MLPA and Affymetrix SNP array 6.0 results in 24 patients underwent array assay

Patient ID	MLPA	Affymetrix human SNP 6.0 Array						Clinical findings								
		
		Gain/loss	Chromo-some band	Start (Mb)	End (Mb)	Size (Mb)	Number of genes	Gender	Age	Seizure	Autistic feature	Dysmorphism or other congenital abnormalities	Micro- cephaly	Low birth weight	Growth delay	Family history
1472	1pter-	loss	1p36.33-p36.32	0.83	3.12	2.29	74	F	7 y 10 m	/	/	hirsutism, hypertelorims, clinodactyly	/	+	/	/

1115	4pter-	loss	4p16.3-p16.1	0.057	9.83	9.77	138	M	6 m	/	/	Greek warrior helmet appearance of the nose, cleft lip/palate, polymicrogyria, thin corpus callosum	+	+	/	/

1483	4pter-	loss	4p16.3-p16.2	0.057	3.38	3.32	58	F	9 m	/	/	Greek warrior helmet appearance of the nose, micrognathia, high-arched palate, dermatoglyphic anomalies	+	+	+	/

1834	4pter-	loss	4p16.3-p16.1	0.057	9.82	9.77	138	F	1 y 3 m	+	/	Greek warrior helmet appearance of the nose, micrognathia, prominent ear, cleft palate, calcification of kidneys, hepatic hemangioma, CHD, dysplasia of atlas	+	+	+	/

1980	4pter-	loss	4p16.3-p16.2	0.056	4.12	4.07	67	F	1 y 2 m	+	/	Greek warrior helmet appearance of the nose, high-arched palate, prominent ear	+	+	+	+

1971	7qter-	loss	7q36.3	157.29	158.82	1.53	7	F	2 y 1 m	/	/	low hair line, hypertelorism, ptosis, prominent ears, hand anomalies	+	/	/	/

339	8pter-	loss	8p23.3	0.021	2.067	2.05	28	F	1 y	/	/	hypertelorism, long philtrum, malformed ears	+	/	/	/

1467	9pter-	loss	9p24.3	0.67	1.54	0.87	5	F	2 y 11 m	/	/	/	+	/	/	/

2268	9pter-	loss	9p24.3-24.1	0.19	7.01	6.82	48	M	3 y	/	/	flat nasal bridge, prominent ears, dermatoglyphic anomalies	/	/	/	/

419	11qter-	loss	11q25	130.33	134.45	4.12	20	M	7 m	/	/	long philtrum, low set ears, micrognathia, thin upper lip, V-shaped mouth	/	+	/	/

1591	11qter -	loss	11q23.3-q25	119.98	132.81	12.82	143	F	2 y 5 m	/	/	light hair color, Hypertelorism, high-arched palate, dermatoglyphic anomalies, CHD	/	/	/	/

1309	15qter-	loss	15q26.2-q26.3	92.79	100.29	7.50	48	M	11 m	/	/	flat nasal bridge, small palpebral fissure, hypertelorism, dermatoglyphic anomalies, clinodactyly, inguinal hernia, polycytic kidney	/	+	/	+

2689	16pter-	loss	16p13.3	0.00077	1.28	1.28	69	F	6 y	/	/	high-arched palate, hypertelorism, prominent ears, thin corpus callosum	/	/	/	/

2498	22qter-	loss	22q13.32-13.33	47.43	49.41	1.97	41	F	3 y	/	/	/	/	+	/	/

1038	3qter+	gain	3q29	194.37	199.38	5.01	67	M	5 y 10 m	+	/	hirsutism, prominent ears, dermatoglyphic anomalies, CHD, cryptorchidism	+	/	/	/

1518	3qter+	/	/	/	/	/	/	M	5 y 11 m	+	+	hirsutism, prominent ears, sparse teeth	/	/	/	/

1729	22qter+	gain	22q13.33	49.32	49.58	0.26	7	F	7 m	/	/	flat nasal bridge, small palpebral fissure, prominent ears, dermatoglyphic anomalies, CHD	+	/	+	/

1704	14qter-	loss	14q32.33	104.53	105.21	0.68	24	M	4 y 6 m	+	/	/	/	/	/	/
										
	17qter-	/	/	/	/	/	/									

390	13pter-	/	/	/	/	/	/	M	1 y	/	/	thin corpus callosum	+	/	/	/
										
	22qter-	loss	22q13.31-q13.33	46.90	49.58	2.68	46									

337	2pter-	loss	2p25.3	0.0028	2.02	2.01	19	F	8 m	/	/	hypertelorism, flat nasal bridge	/	/	/	/
										
	4pter+	gain	4p16.3	0.15	1.19	1.04	24									

1947	10pter-	loss	10p15.3-15.1	0.062	6.22	6.16	48	M	9 m	/	/	hypertelorism, flat nasal bridge, micrognathia, CHD	+	/	/	+
										
	6qter+	gain	6q27	165.60	166.43	0.82	6									

1711	8pter-	loss	8p23.3	0.16	1.91	1.75	17	M	4 y 10 m	/	/	long philtrum	+	/	/	/
										
	7pter+	gain	7p22.3	0.26	1.81	1.55	28									

2126	6pter-	loss	6p25.3	0.33	2.95	2.63	20	M	3 y 3 m	/	/	thin corpus callosum	/	/	/	+
										
	8pter+	gain	8p23.3	0.021	0.58	0.56	7									

2026	7pter-	loss	7p22.3	0.26	2.01	1.76	23	F	1 y 7 m	/	/	ptosis, long philtrum, downturned corners of the mouth	/	/	/	/
										
	12pter+	gain	12p13.33-13.2	0.021	11.07	11.05	202									

+ = positive, / = negative

## Discussion

Since the identification of submicroscopic subtelomeric rearrangements as a cause of idiopathic MR in 1995, testing for subtelomeric abnormalities has become an important clinical evaluation step for the etiological diagnosis of unexplained DD/MR in Western countries [1,4,26-29]. This is the first large-scale study carried out on submicroscopic subtelomeric aberrations in Chinese patients.

### Subtelomeric aberrations identified in Chinese patients

Obtaining an accurate prevalence for subtelomeric aberrations in idiopathic DD/MR is difficult [7]. Using MLPA followed by array analysis, we achieved a detection rate of 5.1% (23/451) in Chinese patients with moderate to severe clinically unexplained DD/MR. This was comparable to most previous reports on other populations. We identified several well-known terminal deletion syndromes in 4p16.3, 1p36, 22q13.3 and terminal 11q. Rare chromosomal terminal aberrations were also found, including deletions in 8p23.3, 7q36.3, 15q26.2-q26.3 and 14q32.3, as well as 22q13 duplication. Of all the subtelomeric aberrations found in Chinese subjects, the most common was 4p16.3 deletion (Wolf-Hirschhorn syndrome), identified in 4/23. In this study, four deleted subtelomeric regions and one duplicated region were smaller than any other previously reported, specifically the deletions in 11q25, 8p23.3, 7q36.3, 14q32.33, and the duplication in 22q13. This information will be helpful for further defining the critical regions that contain candidate DD/MR- associated genes.

### Subtelomeric aberrations smaller than previous reports

#### 11q terminal deletion

**Figure 1:**
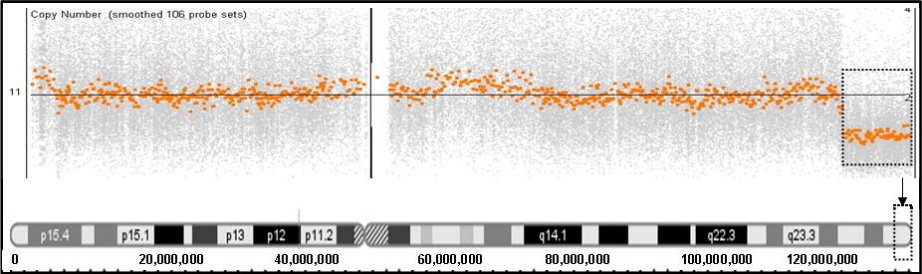
**Deletion in 11q25 shown in Affymetrix human SNP array 6.0**. Affymetrix human SNP array 6.0 assay shows a 4.11 Mb deletion in 11q25, which is indicated with the dashed rectangle.

We found 2 patients (patient 419 and 1591) with an 11q terminal deletion, which is reported to present with DD, short stature, congenital heart disease(CHD), thrombocytopenia, genitourinary anomalies, pyloric stenosis, and ophthalmologic defects. The critical regions of the deletion might correlate with specific clinical phenotypes. Grossfeld et al defined critical regions for 14 individual phenotypes [30]. The smallest critical region (6.8 Mb) they reported extended from D11S1351 (located in 11q24.2) to the telomere, and was associated with four phenotypes, including Paris-Trousseau platelet disorder, undescended testes, pyloric stenosis, and MR. Coldren et al proposed two loci in distal 11q related to global and selective deficits in neurocognitive function [31]. The smallest deletion 11q terminus was reported by Bernaciakin in 2008, which was 5 Mb in size [32]. In our study, patient 419 had a 4.11 Mb deletion in 11q25, spanning from 130.33 Mb to 134.45 Mb (figure 1), which is smaller than any other previous reports. The patient had DD and facial dysmorphism including thin upper lip, V-shaped mouth, micrognathia and low-set ears, but without platelet disorder, undescended testes or pyloric stenosis. Thus, the critical region underlying the phenotype of DD/MR in 11q terminal deletion disorder is probably in the most distal part, within 4.11 Mb of the telomere. The deleted region contains 20 genes, with 14 expressed in human brain. *SNX19 *(sorting nexin-19), *THYN1 *(thymocyte nuclear protein 1), *OPCML *(opioid-binding protein/cell adhesion molecule-like), *VPS26B *(vacuolar protein sorting 26, yeast, homolog of B), *NCAPD3 *(non-SMC condensing II complex subunit D3) and *NTM *(neurotrimin) might be candidate genes, and the encoded proteins are associated with intracellular trafficking, phosphoinositide binding, mitotic chromosome assembly and segregation, outgrowth of neurites, and apoptosis.

#### 8p23.3 deletion

Microscopically visible distal 8p deletions are associated with growth and mental impairment, minor facial anomalies, congenital heart defects, and behavioral problems. Submicroscopic subtelomeric 8p deletion is uncommon, with only a few reported cases [33]. de Vries BB et al reported two cousins with 5.1 Mb of terminal 8p deletion, who presented with mild MR, normal facial appearance, normocephaly and behavioral problems such as unsocialized conduct disorder [34]. We found 1 patient (339) with a very small deletion in terminal 8p, 2.06 Mb to the telomere (in 8p23.3). This one-year-old girl presented with DD, microcephaly and minor facial dysmorphism. Thus, we can define the critical region underlying DD/MR in subtelomeric 8p deletion within the distal 2.05 Mb. This area contains 28 genes, and 12 are known to be expressed in human brain. Among them, *CLN8 *and *DLGAP2 *(discs large-associated protein 2) might be candidate genes. *CLN8 *is involved in lipid synthesis and transportation, and mutations are related to neuronal ceroid lipofuscinoses (NCL), an autosomal recessive neurodegenerative disorder. The product of *DLGAP2 *is a membrane-associated guanylate kinases localized at postsynaptic densities in neuronal cells. This kinase is in a family of signaling molecules found at various submembrane domains, and may play a role in the molecular organization of synapses and in neuronal cell signaling.

#### 7q terminal deletion

A few cases were found to have terminal 7q deletions. The 7q36-qter deletion is usually associated with DD/MR, low birth weight, growth retardation, abnormal skull shape, and some facial dysmorphism like nose malformation, hypertelorism, and ear malformation [35]. This study reports a patient with the smallest deletion (1.53 Mb) detected in terminal 7q to date. She was 2 years of age, and presented with DD, microcephaly, low hair line, ocular hypertelorism, ptosis, and prominent ear and hand malformations. The deleted region was in 7q36.3, spanning from 157.29 Mb to 158.82 Mb, which contains only seven genes. *PTPRN2 *(protein tyrosine phosphatase, receptor type N), *NCAPG2 *(leucine zipper protein 5), *VIPR2 *(vasoactive intestinal peptide receptor 2), *FAM62B *(family with sequence similarity 62) and *WDR60 *(WD repeat domain 60) are expressed in human brain. *PTPRN2 *and *NCAPG2 *might be candidates. The protein encoded by *PTPRN2 *is a member of the protein tyrosine phosphatase (PTP) family. PTPs are signaling molecules that regulate a variety of cellular processes including cell growth, differentiation, mitotic cycle, and oncogenic transformation. NCAPG2 is a non-SMC (structural maintenance of chromosome) subunit that defines condensin II. Condensin complexes I and II play essential roles in mitotic chromosome assembly and segregation. It's reported that microcephalin/MCPH1 is one of the causative genes responsible for the autosomal recessive disorder primary microcephaly. Patients with this disease present with mental retardation and dramatic reduction in head size, and cells derived from these patients contain abnormally condensed chromosomes. More recently, MCPH1 has been implicated in the cellular response to DNA damage, although its exact mechanism remains unclear. In 2008, Wood et al identified condensin-II as a major MCPH1-interacting protein. MCPH1 and condensin II interact in vivo, mediated by the CAPG2 subunit of condensin II [36].

#### 14q32.33 deletion

We identified one patient (1704) with a very small deletion in 14q32.33. Terminal deletions of the 14q are rare. The common clinical features shared by patients with 14qter deletions include mild to moderate DD/MR, microcephaly, high forehead with lateral hypertrichosis, broad nasal bridge, long and broad philtrum, thin upper lip, high arched palate, single palmar crease, and hypotonia [37]. Seizures are absent in most patients with 14qter deletions, but were reported in one case with a 3.2 Mb terminal deletion in 14q32.32 [38]. Early onset intractable seizures usually present in ring 14 rather than 14qter deletions [39]. Patient 1704, a 4-year-old boy, presented with moderate DD and seizures, with no dysmorphism or congenital abnormalities. The terminal 14q deletion found in this patient is the smallest reported to date, spanning from 104.53 Mb to 105.21 Mb, for only 0.68 Mb. This region contains 24 genes, with 15 expressed in human brain. *MTA1 *(metastasis associated protein), *BRF1 *(transcription initiation factor IIIB isoform3), *NUDT14 *(nucleoside diphosphate-linked moiety × motif 14) and *JAG2 *(jagged 2 isoform a precursor) might be candidate genes for DD/MR. They are involved in regulation of transcription, cell proliferation, glycosylation and intercellular signal transduction.

#### 22q13.3 deletion and duplication

We found both deletions and duplications in terminal 22q (patient 390 with 22q13.31-13.33 deletion, patient 2498 with 22q13.32-13.33 deletion and patient 1729 with 22q13.33 duplication). 22q13.3 deletion syndrome is a recognizable malformation syndrome associated with DD, hypotonia, delayed or absent speech, autistic-like behavior, normal to accelerated growth and dysmorphic faces with epicanthal folds, large/dysplastic ears, pointed chin, dolichocephaly and ptosis [40,41]. Anderlid et al refined the critical area to 100 kb in 22q13.33, which contains three genes, *ProSAP2 *(*SHANK3*), *ACR *(acrosin precursor) and *RABL2B *(RAB, member of RAS oncogene family-like 2B) [42]. *SHANK3 *is a good candidate gene, as it is preferentially expressed in the cerebral cortex and cerebellum, and encodes a scaffolding protein involved in the postsynaptic density of excitatory synapses. De novo deletions and mutations of *SHANK3 *have been found in individuals with autism [43-47]. The deleted region in patient 390 was 2.68 Mb in size, extending from 4.69 Mb to 4.96 Mb, containing 46 genes, including the reported critical area. The patient had low birth weight, normal growth and a thin corpus callosum. He was only one year old, so delayed or absent speech or autistic behavior could not be determined. With 1.97 Mb of deletion in 22q, patient 2498 showed absent speech at 3 years of age. The 22q13 duplication is not common, with only a few cases reported. Some clinical features, including intrauterine growth retardation, CHD and other dysmorphism were described [48]. Patient 1729 had some facial dysmorphism, CHD, microcephaly and growth delay. She had a 258 kb duplication, spanning from 4.93 Mb to 4.96 Mb. To our knowledge, this is the smallest reported duplication in the terminal region of 22q. Seven genes reside in the duplicated area, which contains *SHANK3*, *ACR*, *RABL2B*, *MAPK8IP2 *(mitogen-activated protein kinase 8 interacting) and *ARSA *(arylsulfatase A, isoform b). Therefore deletion as well as duplication of critical genes like *SHANK3*, are likely to be associated with common phenotypes like DD/MR, since DD/MR is shared by all patients with 22q13.33 deletion or duplication.

### Subtelomeric duplications

We found pure terminal duplications in 2/451 subjects (0.4%). Subtelomeric pure microduplications are an infrequent cause of MR/MCA with a frequency of approximately 0.5% [49]. We identified 22qter and 3qter microduplications. Patient 1038 showed common features of the dup (3q) syndrome including hirsutism, microcephaly, low-set ears, malformation of hands, cryptorchidism and CHD [50]. He had recurrent afebrile seizures since 3 months of age. A 5.0 Mb duplication in 3q29 (from 194.37 Mb to 199.38 Mb) was detected in this patient, containing 67 genes. Our finding supports the observation made by Battaglia et al. [51], who suggested that the critical region underlying the phenotype of dup 3q is in 3q29.

### Common phenotypes in submicroscopic subtelomeric aberrations

For most subtelomeric rearrangements, a specific phenotype has not been defined, making recognition and selection of patients for such tests challenging in clinical practice. Some common clinical features might be shared by patients with various subtelomeric abnormalities. A five-item checklist of clinical features for pre-selection of patients for subtelomeric rearrangements was proposed by de Vries BB et al, including: 1) family history of MR; 2) prenatal onset of growth retardation; 3) postnatal growth abnormalities; 4) at least two facial dysmorphic features; and 5) at least one non-facial dysmorphic feature and/or congenital abnormality [52]. In our study, for patients with moderate to severe DD/MR with a normal karyotype, those with subtelomeric rearrangements (n = 23) had a higher percentage of microcephaly (47.8% vs. 24.3%), low birth weight (30.4% vs. 7.5%), maternal history of miscarriage (13.0% vs. 2.1%), facial/non-facial dysmorphism (78.3% vs. 50.7%) and other congenital abnormalities (39.1% vs. 14.7%), than those without subtelomeric rearrangement (table 1). Therefore, these might be common features shared by most DD/MR patients with submicroscopic subtelomeric aberrations. Nonetheless, some patients, like patient 1467, with an 874 kb deletion in 9p24.3, only presented with DD and microcephaly. And patient 1704, with a deletion in 14q32.33, had no clinically recognizable dysmorphism.

## Conclusions

This large-scale study reports the detection of submicroscopic subtelomeric aberrations in Chinese patients with DD/MR for the first time. Subtelomeric rearrangements were found in 5.1%. Although benign subtelomeric variations exist [53], most de novo subtelomeric aberrations are considered pathogenic. Further observations of a larger number of patients with similar submicroscopic subtelomeric abnormalities may lead to the recognition of specific phenotypes, and will be helpful in the clinical etiologic diagnosis of DD/MR. More important, fine mapping of aberrations in gene-enriched subtelomeric regions provides essential clues for localizing critical regions, and provides a strategy for identifying new candidate genes associated with DD/MR.

## Competing interests

The authors declare that they have no competing interests.

## Authors' contributions

YW participated in writing the manuscript and patients' recruitment. YJ and XrW had the primary responsibility for protocol development and writing the manuscript. TJ participated in writing the manuscript and carried out the laboratory work concerning the MLPA and data analysis. JW, HW, JL, ZG, XzW and NZ participated in laboratory work concerning the MLPA. JX, YY, LW, ZZ, LT and JQ participated in the patients' recruitment. BC participated in laboratory work concerning the Affymetrix SNP 6.0 array and data analysis. All authors read and approved the manuscript.

## Pre-publication history

The pre-publication history for this paper can be accessed here:

http://www.biomedcentral.com/1471-2350/11/72/prepub
